# Surgical treatment for primary hyperparathyroidism in the elderly: a single-center analysis

**DOI:** 10.1186/1471-2318-11-S1-A64

**Published:** 2011-08-24

**Authors:** L Venturini, F Frezzotti, A Giannella, G Di Rocco, L Fiengo, D Giannotti, S Federici, C Paciotti, G Patrizi, F Pelle, N Sforza, A Redler

**Affiliations:** 1 Department of Vascular and General Surgery, Faculty of Medicine “La Sapienza”, Roma, Italy

## Backgrounds

The suspicion of a hyperparatiroidism is mostly guided by the finding of an increase in serum calcium levels by routine measurements. Primary hyperparathyroidism is a common disease occurring in 0.2 to 0.5% of the population. The incidence in the United States is approximately 100000 new cases per year and increases with age affecting up to 2% of elderly people [1].

## Materials and methods

From January 1995 to December 2009, 172 patients underwent operations for Hyperparathyroidism, 130 of these were Primary Hyperparathyroidism at our Department of General Surgery. Patients were divided into two groups: patients of ≤ 69 years old (Group A) and patients of ≥ 70 years old (Group B). The following variables were studied: demographic characteristics, co-morbidities, preoperative symptoms, laboratory values, operative procedures, postoperative complications and anatomo -pathological findings.

## Results

Group A: 110 patients operated 25 were male, 85 were female with a M:F ratio of 0.3:1. Mean age at admission was 52.4 (SD±12.9). We reported a morbidity rate of 5.4% (6 patients) and a mortality rate of 0%. Group B: 20 patients operated 6 were male, 16 were female with a M:F ratio of 0.25:1. Mean age at admission was 74.2 (SD±3.7). We reported a morbidity rate of 5% (1 patient) and mortality rate 0%.

## Conclusions

Elderly patients with Hyperparathyroidism present a variety of symptoms that are often different from those seen in younger patients. They are more likely to manifest fatigue and psychiatric symptoms that are difficult to distinguish from those due to their age, therefore in the majority of cases the suspicion of hyperparathyroidism is guided by the finding of an increase in serum calcium levels on a routine measurement. If serum calcium level is high or if hypercalcaemia is discovered, measurement of PTH confirms the diagnosis [2,3]. Cervicotomy and parathyroidectomy is still to be considered as the treatment of choice in elderly patients with primary hyperparathyroidism.
